# Emergency peripartum hysterectomy: A great obstetric challenge

**DOI:** 10.12669/pjms.346.13686

**Published:** 2018

**Authors:** Sarah Kazi

**Affiliations:** 1 *Dr. Sarah Kazi, FCPS, MRCOG. Associate Professor, Department of Obstetrics & Gynecology Unit I, Dow University of Health Sciences & Civil Hospital, Karachi, Pakistan*

**Keywords:** Adherent placenta, Emergency obstetric hysterectomy, Atonic uterus

## Abstract

**Objective:**

To determine the prevalence, etiology and complications of emergency obstetric hysterectomy (EOH) at a tertiary care hospital in Karachi.

**Methods:**

This was a retrospective cohort study which was carried out in the Department of Obstetrics & Gynecology Unit II, Civil Hospital Karachi from March 2015 to March 2017. All patients undergoing EOH were included in the study. Data was collected from medical files and labor room registers in accordance with ethical guidelines. Information included demographic characteristics, reasons for EOH, procedure associated morbidity and mortality. Data was recorded on a predesigned pro forma and analyzed using IBM SPSS Statistics for Macintosh, Version 18.0.

**Results:**

Out of 7,968 deliveries in two years, 32 patients under went EOH, (prevalence 4.01 per 1,000 deliveries). The mean age was 30.0 ± 5.2 years. Most women (75%) were multipara. The main mode of delivery was caesarean section (80%), mostly done as an emergency procedure (81%). In 10(30%) patients EOH was performed for uncontrollable hemorrhage due to uterine atony, followed by a morbidly adherent placenta (28%). Morbidity included disseminated intravascular coagulation (DIC) in four patients(19%) and three patients underwent repeat laparotomy for uncontrollable hemorrhage. There were three maternal deaths, giving a case-fatality index of 9.3%.

**Conclusion:**

Uterine atony and morbidly adherent placenta were the main reasons for emergency obstetric hysterectomy (EOH) in our set up.

## INTRODUCTION

Emergency obstetric hysterectomy (EOH) can be a life-saving intervention for cases of severe intra- or postpartum hemorrhage (PPH). Uncontrolled hemorrhage is a leading cause of maternal morbidity and mortality around the globe, particularly in third world countries like Pakistan. A previous study from our institution showed that hemorrhage was the leading cause of maternal death.^1^ Though obstetric hysterectomy was pioneered more than 200 years ago,^2^ it still has a pertinent role in the management of PPH, especially in developing countries where interventional radiology is not readily accessible. Lack of infrastructure, delayed referral also plays an important role in an increased trend for obstetric hysterectomy.

Conservative methods for the management of PPH include use of prostaglandins, balloon tamponade and compression sutures. Introduction of these methods has resulted in a decrease in the incidence of EOH. In developed countries, despite advanced modalities such as uterine artery embolization, EOH remains the mainstay of treatment for intractable hemorrhage. The incidence of EOH varies around the world, ranging from 0.64 to 5.09 per 1,000 deliveries.^3^,^4^

In rural parts of Pakistan deliveries are often performed by untrained birth attendants, which lead to complications such as obstructed labor and uterine rupture requiring EOH. Cases such as these require life-saving EOH, yet the procedure itself is associated with significant maternal morbidity and mortality.

Our study aimed to evaluate the frequency of EOH in a public sector University Hospital in Pakistan, and to determine its associated risk factors and complications. The study may also provide basis for audit of management in cases of intractable hemorrhage and compare our practice with others around the world.

## METHODS

This was a retrospective study conducted from March 2015 to March 2017. All women requiring EOH for uncontrollable major hemorrhage in the department of Obstetrics & Gynecology Unit-II, Civil Hospital, Karachi were included. Civil Hospital Karachi is a tertiary care hospital, with an annual delivery rate of more than 15,000 deliveries. Due to administrative reasons, the department is divided in three units, each unit having its own emergency days. The hospital caters to the needs of both Sind and the neighboring Baluchistan province. We also receive referrals from neighboring hospitals and cater to nearby rural areas. Data was anonymously collected from hospital files and labor room admission registers in accordance with ethical regulations. EOH was defined as hysterectomy done after 24 weeks gestation and within six weeks of delivery.^5^,^6^ Information collected included patient age, parity, the etiology of obstetric hemorrhage, indication for caesarean section, amount of blood loss and adverse events following the procedure(including admission to intensive care or death). We paid particular attention to medical complications such as disseminated intravascular coagulation (DIC) and other end organ damage. Data was entered into a predesigned pro forma and simple descriptive statistics were performed using IBM SPSS Statistics for Macintosh, Version 18.0. Since this was a retrospective study no ethical approval was required.

## RESULTS

During the two-year study period a total of 7,968 deliveries were performed, of which 32 were EOH yielding a prevalence of 4.01 per 1,000 deliveries. Demographic characteristics of these 32 women are presented in Table-I. The mean age was 30±5.2 years. Most women had parity between 2 and 5(75%). Caesarean section was the main route of delivery (81%).

**Table-I:** Demographic characteristics of 32 patients who underwent EOH.

Characteristics	
***Age***	
<20	2 (6.25%)
20-29	4(12.5%)
30-35	16 (50%)
>35	10 (31%)
***Parity***	
1	1 (3.1%)
2-5	24(75%)
>5	7 (21.8%)
***Mode of delivery***	
Vaginal delivery	6 (18.75%)
Emergency cesarean section	18(56.25%)
Elective cesarean section	8(25%)
Total cesarean sections	26 (81.25%)

The indications for EOH. Uterine atony leading to hemorrhage was the most common etiological factor (31.25%), followed by morbidly adherent placenta (28%) as shown in Table-II. The morbidity associated with EOH is shown in Table-III. There was one (3%) renal injury identified during surgery, which was repaired with urologist support. DIC was seen in four (19%) patients requiring massive blood transfusions. Repeat laparotomy was required in 3 (9%) patients for hemorrhage control. There were 3 (9%) maternal deaths among the study population, yielding a case fatality index of 9.3%. These women underwent massive hemorrhage, and ended in DIC despite massive transfusions. Febrile morbidity is another frequently reported complication in the literature,^2^ yet was not noted in our cohort.

**Table-II:** Indications for EOH in 32 patients.

Indication for EOH	
Uterine atony	10 (31.3%)
Placenta previa	6 (18.7%)
Secondary post partum hemorrhage	2 (6.2%)
Uterine rupture	5(15.6%)
Adherent placenta	9(28.1%)

**Table-III:** Morbidity associated with EOH in 32 patients.

Morbidity	
DIC	4 (12.5%)
Urinary bladder injury	1(3.1%)
Repeat laparotomy	3 (9.4%)
Wound infection	2 (6.3%)
Intensive care stay	11 (34.4%)
Death	3 (9.4%)

## DISCUSSION

The average number of deliveries in our unit in Civil Hospital Karachi ranges from 6,000 to 8,000 deliveries per year. During the two-year study period a total of 32 EOHs were performed, yielding a prevalence of 4.01 per 1,000 deliveries. This rate is considerably higher than those quoted in the published literature, i.e. 0.68 per 1,000 deliveries in Saudi Arabia, 0.25 per 1,000 deliveries in Taiwan, 5.09 per 1,000 deliveries in Turkey and 2.7 per 1,000 deliveries in USA.^3^,^4^,^7^,^8^ The reasons for variations in the incidence may be attributed to the standard of care delivered, the differences in obstetric practices across nations and selective reporting by teaching hospitals around the world. This may also be attributed to the fact that this is a hospital based study. Our department follows universal guidelines for management of PPH.

Uterine atony was the most common reason for EOH in this study, followed by morbidly adherent placenta. Atony was also the most common reason for EOH in studies published by Varras and Rabiou et al.^9^,^10^ Conversely, morbidly adherent placenta and placenta previa have been found as the primary etiological causes for EOH in several other studies.^8^,^11^ One study from a private University Hospital reported that morbidly adherent placenta was the most common cause of EOH.^12^ The authors concluded that a rising rate of caesarean sections was responsible for the increased incidence of EOH, resulting in adherent placentas. In another study from a private University Hospital in Sindh, Pakistan, uterine rupture was the leading cause of EOH.^13^ Though the authors reported a rate of 0.42 per 1,000 deliveries, they had four (19%) maternal deaths in their cohort.

It has been observed that rates of EOH due to uterine atony have decreased with increasing use of balloon tamponade and B-Lynch sutures. In this study, all medical and surgical methods were used prior to hysterectomy. B-Lynch sutures failed in three cases and bilateral internal iliac ligation was done in four patients, but proved successful in only three of these. Morbidly adherent placenta was the second leading cause of obstetric hysterectomy in our patient population, with 9(28%) patients undergoing EOH for this reason.

In cases of PPH, it is of utmost importance to make timely and effective decisions for life-saving procedures. Not only does this prevent mortality, in the best-case scenario, but it also reduces associated morbidities such as prolonged surgery and massive blood transfusion, both of which are associated with their own risks. As shown in our study and supported by the previously published literature, caesarean section has been identified as salient risk factor for EOH.^5^ The identification of an adherent placenta in the antenatal period using Doppler ultrasound and magnetic resonance imaging (MRI) is therefore recommended, and should be used to determine the site and degree of placental invasion before hand. A multidisciplinary approach including surgeons, anesthetists, interventional radiologists, midwives, nurses and the blood blank services should be involved.^14^

Despite regular availability of contraceptives and reduced family size world over, there has still been a consistent rise in the rates of caesarean deliveries giving rise to complications like abnormal placentation and uterine rupture, but also the incidence of PPH, giving obstetric hysterectomy more relevance in present day modern obstetric practice.^15^ Though EOH is often considered a life-saving procedure, it can be associated with a significant post-operative burden. Common surgical complications include injury to the urinary tract and other nearby structures, which may be avoided by better surgical training or multi-specialist input involving urological or general surgeons. Vascular surgeons should be involved and major hemorrhage protocols should be activated.

Other adverse events may include wound infection and dehiscence or intestinal obstruction, which may require an emergency laparotomy in the worst-case. Often ignored is the impact on patients’ mental health. For younger women, in particular, post-traumatic depression causes significant morbidity, and adequate psychological support is crucial in providing holistic care.

A near miss event is defined as a woman who nearly died but survived a complication occurring during pregnancy, childbirth, or within 42 days of termination of pregnancy. EOH can be rightly classified as a near miss event.^16^

### Limitations of the Study

As a tertiary hospital based study, rates of EOH were significantly higher than the general population as we receive referrals from neighboring hospitals and cater to nearby rural areas. Furthermore, most women were grossly anemic on arrival and had no prior antenatal records. Researchers at a private University Hospital found a much lower rates of EOH.^12^

### Recommendations

It is important to educate the women regarding birth spacing. Moreover less aggressive maneuvers such as balloon tamponade and compression sutures should be used more frequently, even in an emergency setting, before performing an obstetric hysterectomy. As adherent placenta was noted to be the second most common cause of EOH, it is recommended that this should be diagnosed via imaging in the antenatal period and an elective surgery should be scheduled. In addition, it is important to develop interventional radiology services in all tertiary healthcare facilities in order to prevent avoidable obstetric hysterectomies.

## CONCLUSION

Uterine atony and adherent placenta were the most common reasons for emergency obstetric hysterectomy (EOH).
